# Is fast reversal and early surgery (within 24 h) in patients on warfarin medication with trochanteric hip fractures safe? A case-control study

**DOI:** 10.1186/s12891-018-2126-3

**Published:** 2018-06-26

**Authors:** Leif Mattisson, Lasse J. Lapidus, Anders Enocson

**Affiliations:** 1 Department of Clinical Science and Education, Stockholm South General Hospital, Karolinska Institutet, Unit of Orthopaedics, S-118 83 Stockholm, Sweden; 2 Department of Molecular Medicine and Surgery, Karolinska University Hospital, Karolinska Institutet, Stockholm, Sweden

**Keywords:** Hip fracture, Surgical treatment, Warfarin, Anticoagulation treatment, Intramedullary nailing

## Abstract

**Background:**

Hip fracture patients in general are elderly and they often have comorbidities that may necessitate anticoagulation treatment, such as warfarin. It has been emphasized that these patients benefit from surgery without delay to avoid complications and reduce mortality. This creates a challenge for patients on warfarin and especially for those with trochanteric or subtrochanteric hip fractures treated with intramedullary nailing, as this is associated with increased bleeding compared to other types of hip fractures and surgical methods.

The aim of the study was to evaluate if early surgery (within 24 h) of trochanteric or subtrochanteric hip fractures using intramedullary nailing is safe in patients on warfarin treatment after fast reversal of the warfarin effect.

**Methods:**

A retrospective case-control study including 198 patients: 99 warfarin patients and 99 patients without anticoagulants as a 1:1 ratio control group matched for age, gender and surgical implant. All patients were operated within 24 h with a cephalomedullary nail due to a trochanteric or subtrochanteric hip fracture. All patients on warfarin were reversed if necessary to INR ≤ 1.5 before surgery using vitamin K and/or four-factor prothrombin complex concentrate (PCC). Per- and postoperative data, transfusion rates, adverse events and mortality was compared.

**Results:**

There were no significant differences in the calculated blood-loss, in-house adverse events or mortality (in-house, 30-day or 1-year) between the groups. There were no significant differences in the pre- or peroperative transfusions rates, but there was an increased rate of postoperative transfusions in the control group (*p* = 0.02).

**Conclusion:**

We found that surgical treatment with intramedullary nailing within 24 h of patients with trochanteric or subtrochanteric hip fractures on warfarin medication after reversing its effect to INR ≤ 1.5 using vitamin K and/or PCC is safe.

## Background

In Sweden about 1.8% of the population is on warfarin medication [1]. Despite a current trend in favour of using Novel Oral Anticoagulants (NOAKs), warfarin is still the most widely prescribed oral anticoagulant for indications such as mechanical heart valves, atrial fibrillation or high grade renal insufficiency and thromboembolic disorder [2, 3]. Hip fracture patients in general are elderly and often have comorbidities that may necessitate anticoagulation treatment, such as warfarin. Now days it is recommended that hip fracture patients should be operated without delay to reduce postoperative complications and mortality [4]. This creates a challenge when surgical treatment of the hip fracture is necessary and the patient is on warfarin medication. It is especially relevant for the management of patients with trochanteric or subtrochanteric hip fractures treated with intramedullary nailing, as these fractures are associated with increased bleeding compared to other types of hip fractures and surgical methods [5, 6]. One of the strategies to reduce the bleeding in these patients is to temporary postpone the warfarin administration and then wait until the anticoagulation effect of the warfarin has subsided, measured as a normal (non-therapeutic) level of the International Normalized Ratio (INR) [7]. However, this may take up to 4 days [8] and according to previous literature this delay can explain up to 8% of all hip fracture surgical delays [9]. Another faster strategy is reversing the effect of the warfarin using vitamin K or four-factor prothrombin complex concentrate (PCC). This may allow surgery without major delay [10]. However, little is published in the literature on this topic and most of the authors who have studied the surgical management of patients on warfarin do not differentiate between different types of hip fractures or surgical methods [7, 11].

The aim of the study was to evaluate if early surgery (within 24 h) of trochanteric or subtrochanteric hip fractures treated with intramedullary nailing is safe in patients on warfarin treatment after fast reversal of the warfarin effect.

## Methods

All patients on warfarin medication operated at our institution with an intramedullary nail due to a trochanteric or a subtrochanteric hip fracture from January 2011 to December 2014 were identified. To be included in the study patients should be: > 60 years of age, have sustained an acute non-pathological trochanteric or subtrochanteric femoral fracture due to a low-energy trauma without other injuries demanding acute surgery or causing major bleeding. Only patients operated within 24 h, calculated from the hospital admission to the start of the surgery were selected. Patients with late presentation to the hospital (> 24 h from injury) were excluded.

A 1:1 ratio control group matched for age, gender and surgical implant (long or short nail), operated within 24 h, was identified. These patients were operated at our institution during the same time period due to non-pathological trochanteric or subtrochanteric fractures after low-energy trauma and had no anticoagulation medication at all.

Patients’ records were searched in order to find information including demographic data, medication, pre- and postoperative data and adverse events occurring during the hospital stay. Mortality data was obtained from the Swedish national Cause of Death Register. Follow-up time was 1 year. Calculation of blood-loss was based on the haemoglobin (Hb) level (g/dL) and the estimated blood volume (BV). The latter was calculated according to gender, weight and height using the formulae [12]: BV (l) = height (m)^3^ × 0.356 + weight (kg) × 0.033 + 0.183 for women, and BV (l) = height (m)^3^ × 0.367 + weight (kg) × 0.032 + 0.604 for men. In assumption that the BV on day 2–4 after surgery was the same as that before surgery and that all the red blood cell (RBC) transfusion units contained the same number of cells (a unit of RBC contains approximately 250 ml and 45 g Hb). The loss of Hb (in grams) was then estimated according to the formula: Hb _loss_ = BV × (Hb _adm_ – Hb _fin_) + Hb _trans_. The Hb _loss_ is the calculated total Hb loss (g), Hb _adm_ is the haemoglobin value (g/dL) on admission, Hb _fin_ is the final recorded Hb value (g/dL) on day 2–4 after surgery, and Hb _trans_ is the total amount of haemoglobin (g) in the transfused RBC units before the measurement of Hb _fin_. We finally estimated the Blood loss (BL) using the following formula: BL (mL) =1000 × (Hb _loss_ / Hb _adm_).

Before the operation all patients on warfarin with an INR > 1.5 were reversed to ≤1.5 using vitamin K (Konakion®), four-factor prothrombin complex concentrate (PCC) (Ocplex®) or both. All operations were performed on a radiolucent traction table and spinal anaesthesia was the standard method for anaesthesia. Implants used were a short, or a long, Gamma3 cephalomedullary nail (Stryker Howmedica, Kalamazoo, MI, USA). Cloxacillin 2 g administered within 30 min before the operation was used as antibiotic prophylactics. Starting on the evening after the operation, a minimum of 2 h after wound closure, low molecular-weight heparin (5000 U dalteparin or 4500 U tinzaparin) was given subcutaneously once daily as thromboprophylactics. Thromboprophylactic therapy was continued until INR was on a therapeutic level for the warfarin patients and for 4 weeks for the control patients. No strict transfusion protocol was used, but the patients were given RBC transfusion based on their current Hb level and a cut off level of 10 g/dL was mostly used. However, the decision whether to transfuse or not was always made on an individual basis with consideration of several factors such as ongoing cardiac disease, blood pressure and other factors were taken into consideration in addition to the Hb value. Patients were mobilised the day after the surgery using necessary walking aids and usually allowed full weight-bearing.

### Statistical methods

The Mann-Whitney U-test was used for comparisons of nonparametric variables in independent groups. The Student’s t-test was used for comparisons of normally distributed variables in independent groups. Normality was tested with the Kolmogorov-Smirnov test. Nominal variables were tested by the Chi-square test or Fisher’s exact test. All tests were two-sided. The results were considered significant at *p* < 0.05. The statistical software used was IBM SPSS Statistics, Version 23 for Windows (SPSS Inc., Chicago, Illinois).

## Results

A total of 198 patients were included in the study: 99 patients on warfarin and 99 patients in the matched control group without anticoagulants. Age, gender and implant type were similar between the groups, as these were the matching variables. The warfarin patients in general had an impaired state of health compared with the control patients, displayed as a lower number of patients with ASA class 1–2 (*n* = 5 versus 18, *p* = 0.007) and a higher mean (±SD) Charlson comorbidity index (5.4 ± 1.3 versus 5.0 ± 1.2, *p* = 0.1). The warfarin patients also had a higher mean (±SD) weight (69 ± 14 versus 64 ± 12 kg, *p* = 0.02), but similar height. All patients were operated within 24 h after admission, but the mean (±SD) time to surgery was shorter for the control group (14 ± 5.6 h) compared to the warfarin patients (16 ± 4.8 h) (*p* = 0.04). All patients except one, (a warfarin patient), was operated under spinal anesthesia. There was no difference in the time of surgery between the groups. Details on patient demographics and surgical data are given in Table 1.

**Table 1:** Demographic and surgical data for all patients

	Warfarin patients (*n* = 99)	Control group (*n* = 99)	*P*-value
Age (years), mean (±SD)	86 (7.4)	86 (7.1)	0.8
Gender, female *n* (%)	69 (70)	69 (70)	1.0
Height (cm), mean (±SD)	167 (10)	166 (10)	0.3
Weight (kg), mean (±SD)	69 (14)	64 (12)	0.02
Charlson comorbidity index, mean (±SD)	5.4 (1.3)	5.0 (1.2)	0.1
ASA class 1 or 2, *n* (%)	5 (5.1)	18 (18)	0.007
Fracture type			
Trochanteric, *n* (%)	68 (69)	66 (67)	0.9
Subtrochanteric, *n* (%)	31 (31)	33 (33)	
Implant type			
Short nail, *n* (%)	66 (67)	66 (67)	1.0
Long nail, *n* (%)	33 (33)	33 (33)	
Time to surgery (hours), mean (±SD)	16 (4.8)	14 (5.6)	0.04
Anaesthesia type			
Spinal, *n* (%)	98 (99)	99 (100)	1.0
General, *n* (%)	1 (1.0)	0	
Time of surgery (min), mean (±SD)	65 (16)	64 (14)	0.8

*ASA* = American Society of Anesthesiologists

### Warfarin patients

The most common indications for warfarin treatment were: atrial fibrillation (*n* = 55/99), atrial fibrillation with previous stroke (*n* = 21/99) or previous embolism (*n* = 7/99) (Table 2). One warfarin patient was treated with low-dose ASA (75 mg) and one patient with dipyridamol in addition. No other anticoagulants were used by the warfarin patients. The initial mean (±SD, range) INR of the warfarin patients was 2.5 (±0.6, 1.2–4.4). Before the surgery patients with INR > 1.5 were reversed to ≤1.5 using vitamin K (*n* = 33/99), PCC (*n* = 14/99) or both (*n* = 45/99). No plasma was used for reversing the warfarin effect (Table 2).

**Table 2:** Details on warfarin patients

Indication for warfarin	*n* (%)
Atrial fibrillation	55 (56)
Atrial fibrillation with previous stroke	21 (21)
Previous embolism	7 (7.1)
Mechanical valve replacement	6 (6.1)
Atrial fibrillation with biological valve replacement	4 (4.0)
Atrial fibrillation with ischemic heart disease	3 (3.0)
Previous stroke	3 (3.0)
Methods for reversing warfarin preoperative	*n* (%)
Vitamin K + PCC	45 (46)
Vitamin K	33 (3.0)
PCC	14 (14)
None	7 (7.1)

*PCC* = four-factor prothrombin complex concentrate

### Adverse events and mortality

The total number of adverse events was 58: 27 in the warfarin group and 31 in the control group (*p* = 0.6). The most common adverse event was a urinary tract infection (*n* = 28), followed by a pressure ulcer (*n* = 20) or a pneumonia (*n* = 16), a myocardial infarction (n = 1) in warfarin group and a stroke (n = 1) in control group, no other thromboembolic disorders, such as pulmonary embolism or deep venous thrombosis, were reported in any group. There were no differences in the numbers of the different types of adverse events between the groups (Table 3). The total in-house mortality was 3.5% (*n* = 7/198), the total 30-day mortality 8.1% (*n* = 16/198) and the total 1-year mortality 26% (*n* = 52/198). There were no differences between the groups when comparing in-house, 30-day or 1-year mortality (Table 3). The mean (±SD, range) length of stay was 4.9 (±2.6, 1–15) days for the warfarin patients and 4.9 (±2.6, 1–16) days for the control group (*p* = 0.9). There was no difference in number of the re-admissions within 30 days between the warfarin patients (9.1%, *n* = 9/99) and the control group (16%, *n* = 16/99) (*p* = 0.2).

**Table 3:** Adverse events and mortality for all patients

	Warfarin patients (*n* = 99)	Control group (*n* = 99)	*P*-value
Myocardial infarction, *n* (%)	1 (1.0)	0	1.0
Stroke, *n* (%)	0	1 (1.0)	1.0
Pulmonary embolism, *n* (%)	0	0	
Deep venous thrombosis, *n* (%)	0	0	
Sepsis, *n* (%)	0	1 (1.0)	1.0
Pneumonia, *n* (%)	6 (6.1)	10 (10)	0.4
Urinary tract infection, *n* (%)	14 (14)	14 (14)	1.0
Pressure ulcer, *n* (%)	10 (10)	10 (10)	1.0
Any adverse event, *n* (%)	27 (27)	31 (31)	0.6
In-house mortality, *n* (%)	5 (5.1)	2 (2.0)	0.4
30-day mortality, *n* (%)	9 (9.1)	7 (7.1)	0.8
1-year mortality, *n* (%)	26 (26)	26 (26)	1.0

### Transfusions and blood-loss

The mean (±SD) preoperative (on arrival) Hb was lower in the control group (12.3 ± 1.5 g/dL) compared to the warfarin group (12.8 ± 1.6 g/dL) (*p* = 0.03) as was the mean (±SD) postoperative Hb (9.9 ± 1.4 g/dL) compared to (10.4 ± 1.2 g/dL) (*p* = 0.01). There were no differences in the late (day 2–4) Hb or the calculated blood-loss between the groups (Table 4). The total rate of patients given any RBC transfusion was 65% (*n* = 128/198). There was a greater proportion of control group patients who received postoperative transfusions (71%, *n* = 70/99) compared to warfarin patients (54%, *n* = 53/99) (*p* = 0.02). There were no differences in the pre- or intraoperative transfusion rates, or the mean total number of units given between the groups (Table 4). Four patients, 3 in the warfarin and 1 in the control group, were given plasma postoperatively.

**Table 4:** Blood-loss and transfusions for all patients

	Warfarin patients (*n* = 99)	Control group (*n* = 99)	*P*-value
Preoperative Hb (g/dL), mean (±SD)	12.8 (1.6)	12.3 (1.5)	0.03
Postoperative Hb (g/dL), mean (±SD)	10.4 (1.2)	9.9 (1.4)	0.06
Day 2–4 Hb (g/dL), mean (±SD)	10.7 (1.1)	10.7 (1.0)	0.7
Calculated blood loss (ml), mean (±SD)	1306 (960)	1285 (588)	0.5
Patients given preoperative transfusion, n (%)	5 (5.1)	1 (1.0)	0.2
Patients given intraoperative transfusion, n (%)	8 (8.1)	12 (12)	0.5
Patients given postoperative transfusion, n (%)	53 (54)	70 (71)	0.02
Patients given any^a^ transfusion, n (%)	56 (57)	72 (73)	0.03
Transfusions per transfused patient, mean (±SD, range)	2.8 (2.8, 1–19)	2.8 (1.4, 1–8)	0.2

^a^Patients given transfusion pre- and/or intra- and/or postoperative

## Discussion

The major finding of this study was that patients with trochanteric and subtrochanteric hip fractures on warfarin and operated within 24 h with an intramedullary nail after fast reversal of the warfarin effect displayed no increased risk for adverse events or increased mortality compared to a matched control group of patients without anticoagulant medication.

Now days most treatment guidelines, local or national, advocate early surgery for hip fracture patients [4, 13–17]. However, this endeavor for early surgery creates a challenge when the patient is on warfarin medication. Lawrence et al. in 2016 [18] showed an association between warfarin therapy with prolonged time to surgery (mean 46 h versus 24 h for the control group), and an increased 1-year mortality rate in hip fracture patients. In an observational study by Dettoni et al. in 2011 [19] including 875 consecutive hip fracture patients they reported that the presence of two critical factors: warfarin therapy and a delay in time to surgery, was associated with a significantly higher risk for complications and mortality compared to all other patients. They concluded that the “discontinue drug and delay surgery” strategy is not suitable for hip fracture patients on warfarin therapy. Obviously, the choice of the way to overcome the anticoagulation effect of the warfarin will influence and sometimes delay the time to surgery. Instead of passively wait until the warfarin effect has faded out, a more active approach can be used. Bhatia et al. in 2010 [20] described that using intravenous vitamin K is a safe and effective treatment to avoid delay in the treatment in this group of patients. Despite this statement they still reported at least 2 days (mean 38 h) delay to surgery in the warfarin group. Similarly, Moores et al. in 2015 [21] described that using vitamin K is a safe method but they still had a time to surgery of 36–48 h in their study. Another option is administration of PCC which shows an effect within 30 min that lasts for at least 6 h. The co-administration of vitamin K will give a stable and rapid reversal of anticoagulation and prevent rebound increases in INR [22, 23]. In our study, almost all the warfarin patients were actively reversed preoperatively and the combination of vitamin K and PCC was the most commonly used method. As we found no statistical difference in the calculated blood loss, no increase in transfusion rate and no decrease in Hb level in the warfarin group we conclude that using vitamin K and/or PCC as mentioned above minimize bleeding.

As known from other studies the extra-capsular (trochanteric and subtrochanteric) hip fractures, which are especially studied in this paper, are associated with increased bleeding tendency in comparison with the intra-capsular hip fractures [24, 25]. In addition, other studies found that intramedullary nailing compared with other surgical techniques was associated with an increased rate of RBC transfusions [6, 26, 27]. In 2006 Foss et al. [6] found that the median calculated total peri-operative blood loss in patients that had undergone intramedullary nailing was increased compared to patients that were operated with arthroplasty due to a hip fracture (1.86 L versus 1.30 L, respectively). Similarly, in a recent study by Cohn et al. in 2017 [28] they found significantly lower preoperative Hb in patients undergoing intra medullary nailing (extra-capsular fractures) and a greater blood loss in the warfarin group compared with a control group (1.22 L versus 1.19 L) but this was not statistically significant. In contrast we found no difference in the calculated blood-loss when comparing warfarin patients with the control patients. One reason could be that we used a more active approach when reversing the warfarin effect that resulted in a pronounced shorter time to surgery in comparison with Cohn et al. (mean 16 versus 47 h). Cohn et al. furthermore reported that their warfarin patients were delayed to surgery (mean 29 versus 47 h) and had a longer hospital stay (mean 5.6 versus 8.6 days) when comparing control patients with warfarin patients. This indicates the superiority of using vitamin K and/or PCC to reduce the INR level to ≤1.5 and operate these patients without delay within 24 h.

We found no difference in the frequency of adverse events or mortality up to 1 year between warfarin patients and the control group. This is in line with Cohn et al. but unfortunately, they do not report mortality beyond the discharge from the hospital.

Somewhat to our surprise we found a lower postoperative RBC transfusion rate in the warfarin group. We speculate that this is because a prolonged effect of the reversing agents administered preoperatively. We found a high transfusion rate in both the warfarin and control cohorts. However, this is in line with other published literature such as Desai et al. [5] who found that 52% of intertrochanteric fractures treated with an intramedullary nail were transfused. In addition Fazal et al. [29] recently reported a transfusion rate of 53% in 79 patients who underwent nailing surgery and among those the transfusion rate was 72% for the 32 patients that were treated with a long nail. Finally, Boone et al. [30] reported a transfusion rate of 57% and 40% for long and short nails respectively.

One obvious limitation of this study is its retrospective design. Ideally this topic should be investigated within the context of a randomized controlled trial. However, the current knowledge and opinion would hardly ethically allow a study that compared early and late surgery in these fragile patients. Another limitation is that we lack information on late complications (after hospital discharge). However, this is to some extent compensated for by that we do have accurate mortality data up to 1 year after surgery. Most of the patients in our cohort had relatively benign indications for warfarin, such as atrial fibrillation. Therefore, the safety of active warfarin reversal in patients on warfarin for reasons other than atrial fibrillation remain unaddressed in this study. The major strengths of the study are the large and homogenous groups, both the study group and the control group. This limits the influence of confounding factors on the results.

## Conclusion

We found that the surgical treatment with intramedullary nail within 24 h for patients with trochanteric or subtrochanteric hip fractures on warfarin medication after fast reversing its effect to INR ≤ 1.5 using vitamin K and/or PCC is safe.

## Abbreviations

ASA class: American Society of Anesthesiologists classification
BL: Blood loss
BV: Estimated blood volume
Hb _adm_: The haemoglobin value on admission
Hb _fin_: The final recorded Hb value
Hb _loss_: The calculated total Hb loss
Hb _trans_: The total amount of haemoglobin (g) in the transfused RBC units before the measurement of Hb _fin_
Hb: Haemoglobin
INR: International normalized ratio
NOAKs: Novel oral anticoagulants
PCC: Prothrombin complex concentrate
RBC: Red blood cell
